# Can Sibling Sex Ratios Be Used as a Valid Test for the Prenatal Androgen Hypothesis of Autism Spectrum Disorders?

**DOI:** 10.1371/journal.pone.0141338

**Published:** 2015-10-23

**Authors:** Keely Cheslack-Postava, Ezra Susser, Kayuet Liu, Peter S. Bearman

**Affiliations:** 1 Department of Psychiatry, Columbia University, New York, New York, United States of America; 2 Department of Epidemiology, Columbia University, New York, New York, United States of America; 3 New York State Psychiatric Institute, New York, New York, United States of America; 4 Department of Sociology, UCLA, Los Angeles, California, United States of America; 5 Interdisciplinary Center for Innovative Theory and Empirics, Columbia University, New York, New York, United States of America; The George Washington University, UNITED STATES

## Abstract

**Background:**

Sibling sex ratios have been applied as an indirect test of a hypothesized association between prenatal testosterone levels and risk for autism, a developmental disorder disproportionately affecting males. Differences in sibling sex ratios between those with and without autism would provide evidence of a shared risk factor for autism and offspring sex. Conclusions related to prenatal testosterone, however, require additional assumptions. Here, we used directed acyclic graphs (DAGs) to clarify the elements required for a valid test of the hypothesis that sibling sex ratios differ between children with and without autism. We then conducted such a test using a large, population-based sample of children.

**Methods:**

Over 1.1 million subjects, born in California from 1992–2007, and identified through birth records, were included. The association between autism diagnosis, determined using the administrative database of the California Department of Developmental Services, and the sex of the subsequent sibling was examined using generalized estimating equations. Sources of potential bias identified using DAGs were addressed.

**Results:**

Among male children with autism, 52.2% of next-born siblings were brothers, versus 51.0% for unaffected males. For females with autism, 50.2% of following siblings were brothers versus 51.2% among control females. The relative risk of a subsequent male sibling associated with autism diagnosis was 1.02 (95% confidence interval: 0.99, 1.04).

**Conclusions:**

In a large, population-based sample we failed to find evidence suggesting an excess of brothers among children with autism while controlling for several threats to validity. This test cannot rule out a role of any given exposure, including prenatal testosterone, in either risk of autism or offspring sex ratio, but suggests against a common cause of both.

## Introduction

Autism is a neurodevelopmental disorder involving impairments in social interaction, communication, and restricted or repetitive behaviours, noted for its marked but unexplained male preponderance. One well developed hypothesis proposed to explain this excess of male cases focuses on the idea of an ‘extreme male brain’—an exaggeration of typical sex differences shaped by prenatal factors, in particular, testosterone levels [1]. Under certain conditions discussed later, this hypothesis (henceforth termed the testosterone hypothesis) predicts that the sibling sex ratio will be different for children with autism compared with non-autistic children, that is, there will be an excess of male births among siblings of autistic children.

The current study had two goals. The first was to clarify the elements required to conduct a valid test of whether the sibling sex ratio differs for children with and without autism, consistent with the presence of a shared antecedent of both autism and sibling sex ratio. As detailed below, other causal structures may also give rise to an observed association between autism and sibling sex ratio. Our goal was to conduct a test such that an observed association would imply a shared antecedent factor. The second was to conduct such a test using a large, population-based sample of children with and without autism.

Some previous reports have indeed suggested an excess of male births among siblings of children with autism, and among siblings of children with related neurodevelopmental disorders [2–4]. Related disorders are relevant because male predominance is also evident in other disorders currently grouped as Autism Spectrum Disorder (ASD) in DSM-V, in disorders co-morbid with autism, and in many other neurodevelopmental disorders (i.e.[5–8],). These previous reports on sibling sex, however, had significant limitations. Generally they used relatively small and selected samples, and/or made comparisons with summary population values for the proportion of male births rather than with controls ascertained in a similar manner as cases, and/or did not meet some of the other criteria described below as conditions for a valid test. In the largest study thus far, significantly more males (58.5%) than expected (51.4%) were reported among 513 siblings of individuals with autism assessed at Danish clinics from 1960–1985 [3]. Siblings included were those reported at the time of clinical assessment, and expected sex ratio was based on the contemporary Danish live-birth ratios. Similar results were reported for developmental language disorder [4]. A much larger Danish register-based study was subsequently conducted, and did not find a significant difference in the sex ratio of siblings of people with ASD (50.8% male) relative to the live birth sex ratio from the corresponding years (51.3% male). Some differences for ASD subtypes were reported. Specifically, siblings of subjects with Asperger syndrome were less likely than expected to be male, whereas siblings of subjects with atypical autism were more likely to be male [9].

### Conditions for a valid sibling sex ratio test

Legitimate questions could be raised about the explanatory power of the sibling sex ratio test for the testosterone hypothesis. For example, with longitudinal measurements of maternal and foetal testosterone, one might construct a more direct test of the testosterone hypothesis. Although such designs have been explored, they have not yet been used in full studies, and are beyond the scope of this paper. Therefore we focus here only on conducting a valid sibling sex ratio test.

Three related premises are required for the use of the sibling sex ratio as a test of the testosterone hypothesis. The first premise is that children with autism and their siblings share a common cause that makes them more likely to be male than female. Thus, the predicted difference in sibling sex ratio of persons with versus without autism is due to confounding of the association between autism in one child and sex of their siblings rather than direct causation of sibling sex by autism. The second premise is that the common cause is prenatal testosterone. This premise is supported by some prior literature suggesting that higher prenatal testosterone levels could be shared by siblings and could increase the probability of offspring being male [10]. While the sex of a given individual is determined by an XX or XY chromosomal karyotype, the sex ratio of offspring at birth is dependent on both the ratio of male to female embryos at conception and their relative survival during gestation [11]. Previous applications of the sibling sex ratio test with regard to autism have focused on prenatal testosterone as an exposure hypothesized to influence this ratio. The observation of an association between autism and sibling sex ratio would also be compatible, however, with the presence of some other common cause. For example, maternal anxiety disorders [12] and exposure to stressful events [11] have been associated with variation in offspring sex ratios. The test presented here could equally be applied as evidence for or against their association with autism under corresponding assumptions. Our contribution here is to clarify the elements required for such a test and not to evaluate the evidence for or against any specific mechanisms hypothesized to affect sex ratios. The third premise is that autism in one child does not itself affect the probability that siblings are male versus female. A direct effect of autism on sibling sex would not represent a common cause shared by the siblings as postulated in the first premise. Although actually a corollary of the first premise, this third premise is worth noting explicitly because it is sometimes overlooked.

In order to explain the import of these premises for a valid test, we begin by portraying the different causal relationships that could underlie an observed association between autism and sibling sex ratio. We use directed acyclic graphs (DAGs), an increasingly common method for illustrating assumptions about causal associations between variables and determining how non-causal statistical associations may arise, explained in depth elsewhere [13;14]. In brief, a DAG is a diagram of assumed causal relationships in which variables are connected by arrows that point in one direction only; from cause to effect. In a DAG the absence of an arrow between two variables assumes that there is no direct causal effect between them. But there may still be a statistical association between the two variables, if they are connected by a series of arrows that form an “unblocked” pathway. An unblocked pathway is one that meets two conditions. First, it does not contain a “collider”, that is, a variable with two arrows pointing into it, i.e. a common effect of both variables. Second, given there is no collider, no variable in the path has been conditioned on (through adjustment, stratification, or restriction of the sample). Further properties of DAGs will be introduced when relevant in the scenarios below.

**Fig 1:**
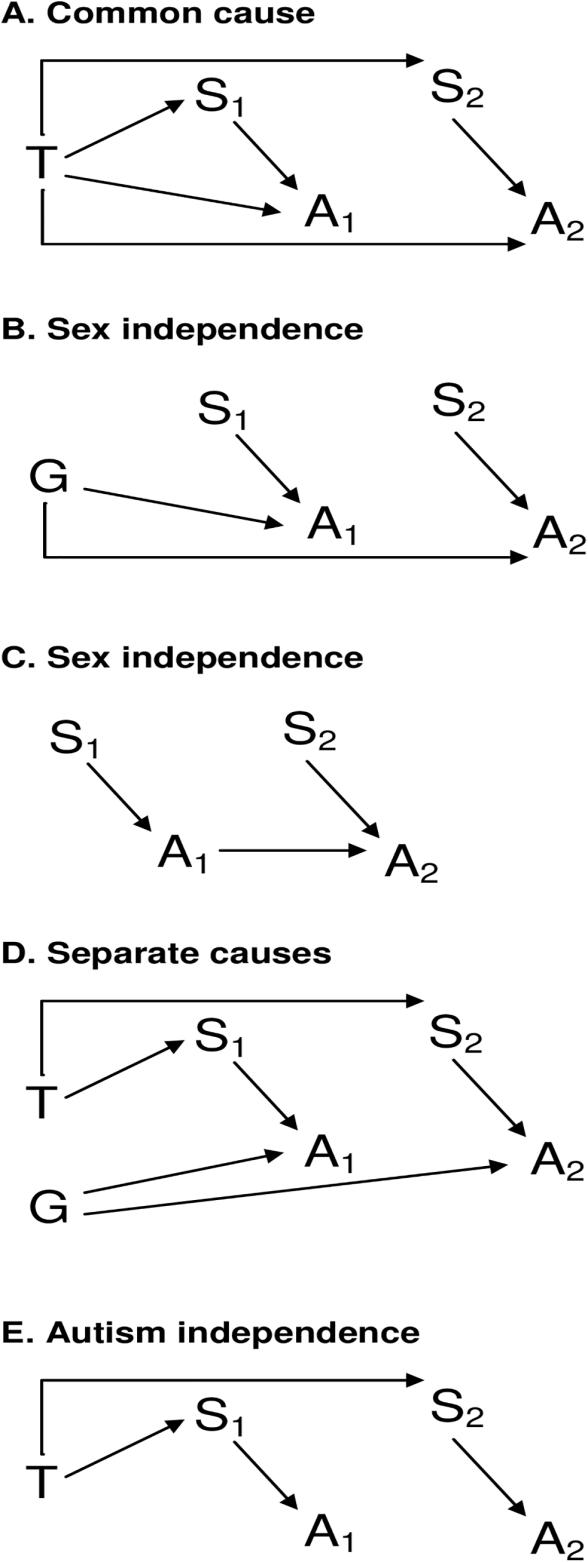
Possible causal structures underlying a hypothesized association between autism and sibling sex. In the common cause scenario (A), an association arises because risk of autism and sex of each child share a common antecedent (for example, maternal testosterone levels). In the sex independence scenarios (B, C), risk of autism in siblings shares a common cause (B), or observation of autism in one sibling directly influences observation of autism in the other (C), but sex is independent. In the separate causes scenario (D), both sex of siblings within a given family and risk of autism in those children are correlated, but the association arises through separate antecedent factors. In the autism independence scenario (E), sex of siblings shares a common cause but risk of autism does not. Depending on method of analysis, an association between whether autism is diagnosed in one child (A_1_) and sex of the sibling (S_2_) could potentially be observed under any of these scenarios.

Five basic scenarios are depicted using DAGs in Fig 1. Each involves two characteristics, sex (S) and whether or not the person has an autism diagnosis (A), observed in each of two siblings (denoted by subscripts). Two siblings only are depicted for simplification but the concepts generalize to greater numbers of siblings. In each scenario, arrows from S_i_ to A_i_ indicate direct causal influence of “sex” on autism diagnosis, for example through a gender bias in case ascertainment [15], X-linked genes [16], or a “female protective effect”[17].

Fig 1A represents the “common cause” scenario that invokes the premises of the sibling sex ratio test described above. T represents prenatal levels of maternally produced testosterone. Individual levels of serum testosterone are correlated over time in women of reproductive age [18]. Arrows from T to S and A in each child depict the joint hypothesis that maternal prenatal testosterone levels affect both sex and risk of autism in each child.

Fig 1B shows one kind of “sex independence” scenario. A factor G is associated with risk of autism in each child, but that factor is unrelated to observed sex. G for example could be parental genotypes or a stable perinatal or environmental factor. In the scenario depicted, there is no association between A_1_ and S_2_ in a population, because the path between them is blocked at the collider A_2_. An artifactual association could be created, however, by study designs that are often employed. In a study design where a study subject from each family is selected based on having autism (i.e., all A_1_ have autism), it will then follow that the siblings of these study subjects (i.e. A_2_) are selected as *less* likely than by chance to have autism. This is despite the sibling recurrence risk of autism, and will occur because persons in the population with autism have been disproportionately designated as “subjects” rather than “siblings”. This is referred to as “conditioning on a collider” and unblocks the path through A_2_, creating an unblocked path from A_1_ to G to A_2_ to S_2_ in Fig 1B. A statistical association between autism and sibling sex ratio may be observed as a result of opening this path, but such an association will be artifactual, that is, attributable to selection bias in the study design [19].

Fig 1C shows another “sex independence” scenario. An example of this scenario would be when observed autism in one child affects observed autism in the second child directly (arrow from A_1_ to A_2_) through dynamics of ascertainment. The potential to observe association between A_1_ and S_2_ could arise similarly as in scenario 1B, described above.

Fig 1D shows a “separate causes” scenario. Separate factors influence sex and autism risk in all children. This diagram would apply for example if it was in fact the case that maternal testosterone influences probability of producing a male or female zygote (S), but does not affect autism risk; however other factors shared between siblings (G) do influence autism risk.

Yet another potential scenario of “autism independence” is depicted in Fig 1E. In this scenario, there is a shared cause for S in each child, but not for A. In scenarios 1D and 1E, an association between A_1_ and S_2_ would be observed due to the unblocked path from A_1,_ to S_1,_ to the shared cause, T, of sex in both children, to S_2_.

**Fig 2:**
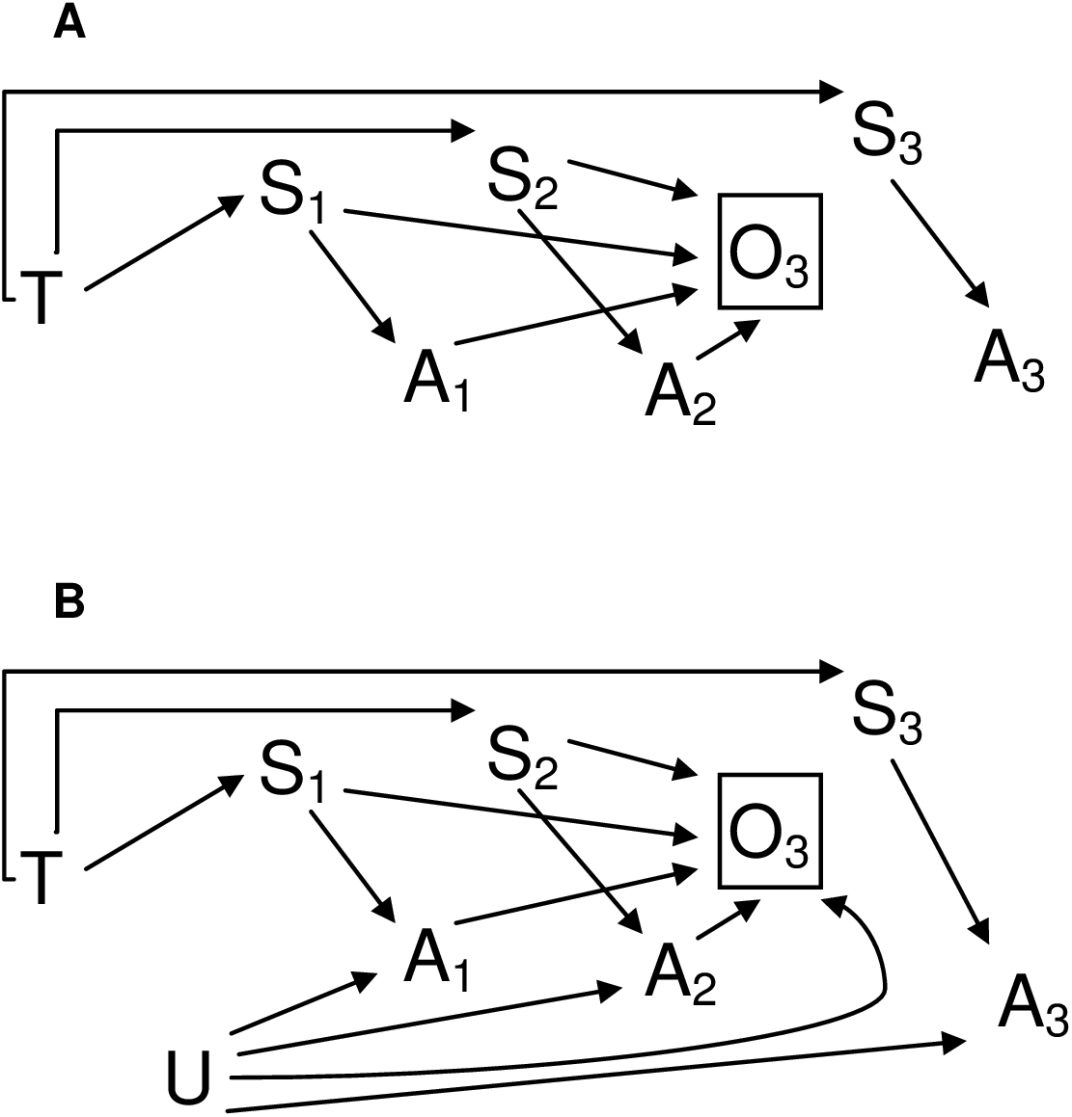
Possible causal structures underlying a selection bias induced association between autism and sibling sex. Preferences regarding sex composition may affect future fertility decisions, and hence observation (O) of additional siblings. This is represented here by arrows from sexes of child 1 and 2, S_1_ and S_2_, to O_3_, the observation (or not) of a third child. If either autism in previous children (A_1_ and A_2_), or shared factors associated with autism (U; ex. parental age at the start of childbearing) also affect future fertility, selection bias may result. This is due to *de facto* conditioning on O_3_ (only children who are conceived and born may be observed), opening a blocked path between autism and child sex. Conditioning on the sex of prior children will block these paths. A box drawn around a variable represents conditioning on it, which, unless it is a collider, blocks any paths through it.

The proposed idea behind examining sibling sex ratios in cases with autism is to infer the type of association shown in Fig 1A. Therefore, a sibling sex ratio test should show an association between autism in the individual selected as the “subject” and sex of subject siblings under the scenario depicted in Fig 1A, but not those shown in Fig 1B, 1C, 1D and 1E. Two additional situations may pose threats to the validity of the test by inducing an observed association under scenarios other than 1A. First, the presence of any time stable factor other than testosterone which is causally associated with both child sex and risk of autism diagnosis will introduce confounding. Second, as illustrated in Fig 2, a type of selection bias could occur if the decision to have additional children is influenced by both the sex composition of prior children (i.e. to fulfil a preference for children of each sex) and by autism diagnosis in prior children (i.e. parents of healthy children may be more likely to have additional children than are parents of children with an illness or disability). Needless to say, only children who are actually born can be observed and included in a study. Therefore, if the sex composition and autism diagnosis of children within a family jointly affect whether or not additional siblings are born into that family (denoted by “O” in Fig 2), then autism in one child, A_n_ and sex of the next child, S_n+1_, will become associated, following the unblocked path from A_n_, through the collider, O_n+1_, to S_n_, to T, to S_n+1_ (Fig 2).

Conditions to ensure that an association between autism in the individual selected as the “subject” and sex of subject siblings will be observed if the “true” underlying scenario is that depicted in Fig 1A, but not if it is one of those shown in Fig 1B, 1C, 1D and 1E; or in Fig 2A and 2B (which are extensions of scenarios 1D and 1E), are summarized in Table 1.

**Table 1:** Summary of criteria for a valid sibling sex ratio test.

*Criterion and justification*	*Scenario(s) which criterion is required to rule out*
**Condition on sex of the subject**	
*Controlling for subject sex (S*_*1*_ *) blocks the only open path between A*_*1*_ *and S*_*2*_ *in Fig 1D and 1E, whereas in Fig 1A they remain associated.*	D, E
**Do not condition or select on autism in subjects**	
*Induces association by opening path blocked by a collider at A*_*2*_. *Preferentially selecting a subject with autism from all affected families will have this effect by making siblings less likely than random to have autism*.	B, C
**Control for sex composition of previous children**	
*Preferences regarding sex composition may affect future fertility decisions, and hence observation of additional siblings. If either autism in previous children, or factors associated with autism (ex. parental age) also affect future fertility, selection bias may result (Fig 2). Controlling for the sex of the subject and preceding children blocks the open paths through which this biased association would flow.*	D, E
**Consider sex of next sibling only**	
*Follows from previous item; otherwise sex composition of previous children will be incompletely controlled*.	D, E
**Control for potential confounders**	
*Required to attribute an observed association to prenatal testosterone (or any other specifically hypothesized factor; versus alternative confounding factor)*.	B, C, D, E

## Methods

### Data Structure

Sibships were identified from data on all births in the state of California from the years 1985–2007, obtained from state Birth Master Files (California Department of Public Health, www.cdph.ca.gov). Infants known to have died before age 1 were excluded. Full siblings were identified by matching on: child’s last name; mother's first name, maiden name, birth place, race, and Hispanic ethnicity; and father's race and Hispanic ethnicity. Scrambled versions of all names were used to protect privacy. Consistency of the matches was checked against mother's birth year and father's birth year derived from mother's age and father's age on the birth record.

### Study subjects

The selection of study subjects is outlined in Table 2. Children born from 1992–2007 comprised the total pool of potential study subjects. Children were excluded if they were not linked to one or more siblings. Families including twins or higher multiple births were excluded. Additionally, children were excluded due to possible erroneous sibling matches if the birth certificate maternal date of last live birth was discordant with prior linked sibling’s date of birth or if any members of the linked sibship included mismatched sequences based on maternal number of live births from birth certificates versus children’s birth dates. Finally, children were excluded if one or more preceding sibling (based on reported number of prior maternal live births) was missing from the data structure, which would preclude control for existing sex composition of the family. Among the children who remained eligible to be study subjects after these exclusions, we selected as our study subjects the 1,192,219 children who had a subsequent sibling observed in the data set (i.e. data on “outcome” was available, see below) and who had data available on all covariates. Although children born from 1985–1991 were not eligible to be subjects because information on autism diagnosis (“exposure”, see below) was only available beginning in 1992, they could still contribute information about family composition.

**Table 2:** Identification of eligible subjects from total population of California births, 1992–2007 and percentage male among included and excluded births.

	N	% male	Sex ratio
Underlying population (full birth cohorts^a^)	8,716,034	51.1	1.05
Linked to one or more siblings	3,864,372	51.1	1.05
Eligible for inclusion^b^	2,171,764	51.4	1.06
Exposure and complete covariate information available	2,148,141	51.4	1.06
Have a next sibling observed	1,192,219	51.6	1.06

^a^ Excluding infant deaths and observations missing sex.

^b^ Exclusion criteria included members of families with multiple births, discordant date of last birth data, one or more preceding sibling births missing from data set, or members of families with discordant sequences (birth date versus maternal parity).

### Exposure

The independent variable (“exposure”) for analyses was presence/absence of an autism diagnosis in a study subject. Children diagnosed with autism were identified using client records of California’s Department of Developmental Services (DDS; www.dds.ca.gov) from July 1, 1992 through June 30, 2011. DDS provides services to persons with autism and other developmental disabilities such as mental retardation, epilepsy, and cerebral palsy; other autism spectrum disorders including Asperger syndrome and pervasive developmental disorder-not otherwise specified (PDD-NOS) alone do not qualify a person for services. It has previously been estimated that the DDS system includes 75–80% of children with autism in the state [20].

### Outcome

The dependent variable (“outcome”) was sex of a subject’s next sibling. Sex of children at birth was determined from the birth master file. Information on sibling sequence was derived within matched sibling sets from birth dates and maternal reported total number of previous live births for each child.

### Statistical Analyses

In order to determine the association between autism diagnosis and sex of the next sibling, while accounting for potential correlation between subjects from the same family, we fit a series of log binomial models using generalized estimating equations with an exchangeable correlation structure and robust variance estimates. In each of these models, autism in the subject was the independent variable, and sex of the next sibling was the dependent variable. In addition to an unadjusted model, models were fit to address concerns enumerated in Table 1. These included models: a) adjusted for subject sex; b) adjusted for potential confounders (maternal and paternal age; maternal race, education, birthplace, parity and payment for delivery using Medi-Cal); c) adjusted for number and sex composition of children to date (categorized as all male, all female, or both); and d) including a product term between subject autism and subject sex, in order to derive separate estimates for male and female subjects. Difference in the male and female estimates was assessed using the product term p-value. All analyses were conducted using Stata version 11.2 (StataCorp LP, College Station, TX). Approval for this study was given by the institutional review board of Columbia University with a waiver of informed consent. Subject information was anonymized prior to analysis.

## Results

The percentage of the population that was male in each step of subject selection is given in Table 2. 51.1% of births in the underlying cohort were male, and this proportion held among those births that matched to one or more siblings. The births that met eligibility criteria for inclusion with regard to data quality and completeness for sibling sequence within families, and had complete covariate data, were 51.4% male. Finally, the subset of those births where a next sibling (“outcome”) was observed were 51.6% male.

Results for analyses showing the association of autism with sex of next siblings are presented in Table 3. 51.9% of study subjects with autism and 51.1% of study subjects without autism had a brother as the next sibling (unadjusted RR [95% CI] = 1.02 [0.99, 1.04]. This estimate of association was essentially unchanged after adjustment for subject sex (RR = 1.02), potential confounders (RR = 1.02), or the number and sex composition of previous children (RR = 1.02). For male and female study subjects considered separately, results for unadjusted analysis were RR (95% CI) = 1.02 [1.00, 1.049]; p>0.05 and RR = 0.98 [0.92, 1.04] respectively. The model including a product term between subject’s sex and autism diagnosis (p = 0.19) did not provide evidence that male and female subjects varied in their associations between autism diagnosis and sex of the subsequent sibling.

**Table 3:** Relative risks that next sibling is male among subjects with versus without Department of Developmental Services (DDS) autism diagnoses among California births, 1992–2003.

	N cases	% male (sex ratio), next sibling of ASD cases	% male (sex ratio), next sibling of non-cases	RR [95% CI]	p_int_
Unadjusted	6690	51.9 (1.08)	51.1 (1.04)	1.02 [0.99, 1.04]	
Adjusted for sex of subject				1.02 [0.99, 1.04]	
Adjusted for potential confounders^a^				1.02 [0.99, 1.04]	
Adjusted for potential selection factors^b^				1.02 [0.99, 1.04]	
Male subject	5598	52.2 (1.09)	51.0 (1.04)	1.02 [1.00, 1.05]^c^	0.19
Female subject	1092	50.2 (1.01)	51.2 (1.05)	0.98 [0.92, 1.04]	

^a^ Paternal age; and maternal age, race, education, birthplace, Medi-Cal, and parity.

^b^ Number and sex composition (girls only, boys only, both) of previous-born, living children.

^c^ p>0.05.

## Discussion

The report here is to our knowledge the most rigorous exploration to date of whether autism in a child is associated with male sex in his/her siblings. Sibling sex ratios have been proposed as a test of hypotheses about the role of prenatal testosterone in influencing the development of autism, and we challenge here how they can be validly used for this purpose. We compared 6,690 children who had autism with controls drawn from the same population, and found no evidence that the two groups differed with respect to the next sibling being male (adjusted RR = 1.02, p>0.05).

Our results are concordant with those of the only prior large-scale study to examine sibling sex-ratios in autism [9], but in contrast to previous reports of such an association, for autism [3] or for ‘male-biased’ disorders including autism [2]. Possible explanations for these differences in prior studies include chance findings in smaller samples; selected samples; and comparison to sex ratios derived from overall populations rather than comparably selected controls (see [21] for an example where this makes a substantive difference).

The sibling sex ratio test conducted here is not a direct test of the hypothesis that maternal testosterone affects autism. Rather, it is a test for evidence of a shared factor influencing both autism and offspring sex ratio. As such, the sibling sex ratio test conducted here does not support the hypothesis that any shared factor (including maternal testosterone) affects both autism and sex of offspring. Three alternative hypotheses would be consistent with the observations made here: 1) maternal testosterone influences autism but not sex in offspring; 2) maternal testosterone influences sex but not autism in offspring; 3) maternal testosterone influences neither sex nor autism in the offspring. We note that this reasoning would equally apply to any other hypothesized shared cause of autism and offspring sex ratio. With regard to the testosterone hypothesis, a fourth explanation, instability of maternal testosterone levels over time, would allow for causal effects of maternal testosterone on both child sex and risk of autism, but posit sufficient variation in within-woman levels over time so that exposure levels and associated outcomes between offspring are uncorrelated. Longitudinal evidence [18] as well as a role of genetic factors in determining levels [22;23] suggests a stable component, but data here cannot eliminate this explanation. However, it should be noted that this would negate the hypothetical utility of a sibling sex ratio test. Similar consideration of temporal variability should be given to any other hypothesized shared exposure for which this test would be applied.

Limitations should be noted. The study used administrative data, and neither diagnosis nor parentage was verified using clinical or laboratory methods; however, several exclusion criteria were applied to reduce the probability of including incorrectly matched siblings. Meanwhile, sex of children is unlikely to be misclassified. This study pertains to children with a diagnosis of autism, and may not reflect other ASD classifications which are not eligible for services through DDS. As with any study, there exists the potential for confounding by unmeasured factors. In order to diminish a true association between autism and subsequent male siblings, a factor would have to be such that levels associated with higher risk of autism diagnosis were associated with lower probability of subsequent male births. We cannot rule this out but note that adjustment for recognized potential confounders made virtually no difference in estimates of association.

The findings presented here call attention to several key issues in interpreting results from epidemiologic studies with regard to sibling sex ratios. First, relatively small studies, such as those that have previously been cited as confirmatory evidence for this explanation of autism and other disorders [24–27], are prone to statistical imprecision. The findings that make it into the literature are most likely to be those that both reach or approach statistical significance (as defined by p-values <0.05), and by extension, those that overestimate the magnitude of any true association. Second, very large studies, including the one reported here, are prone to findings of statistically significant so-called “tiny effects” [28] which are difficult to interpret both with regard to clinical or etiologic relevance and given the omnipresent possibility of residual bias. Even so, the very low magnitude of association between autism and sibling sex reported here failed to reach the traditional benchmark of “statistical significance”. Finally, no matter what the study size, sources of potential bias should be recognized. Adequately addressing such potential bias requires using a control population ascertained in a comparable manner to cases, and cannot be accomplished through a simple comparison to overall population statistics [24–28]. We suggest that future studies examining sibling sex ratios in autism spectrum disorders, or any outcome, should use large, population-based sources of data and rigorous methods to minimize bias.

In conclusion, our results from a large California population do not provide evidence that the sex of a child’s next sibling is associated with whether or not that child has an autism diagnosis, and do not support the joint hypothesis that any common factor (including prenatal testosterone levels) is causally related to both sex and risk of autism among offspring.
